# Periarticular analgesic injection containing a corticosteroid after total hip arthroplasty may prevent deep venous thrombosis: a retrospective comparative cohort study

**DOI:** 10.1186/s12891-020-03879-x

**Published:** 2021-01-06

**Authors:** Akira Hashimoto, Motoki Sonohata, Hirohito Hirata, Shunsuke Kawano, Shuichi Eto, Masaya Ueno, Masaaki Mawatari

**Affiliations:** 1 Department of Orthopaedic Surgery, Faculty of Medicine, Saga University, Nabeshima 5-1-1, 849-8501 Saga, Japan; 2 Research Center of Arthroplasty, Faculty of Medicine, Saga University, Nabeshima 5-1-1, 849-8501 Saga, Japan

**Keywords:** Periarticular analgesic injection, Total hip arthroplasty, Corticosteroid, Anti-inflammation, D-dimer, Triamcinolone acetonide

## Abstract

**Background:**

Of late, periarticular analgesic injection (PAI) has become a common alternative treatment for pain following total hip arthroplasty (THA). However, the systemic effects of PAI containing corticosteroids in patients subjected to THA have not been investigated. This study evaluated the analgesic efficacy and systemic effects of PAI containing a corticosteroid in patients subjected to THA.

**Methods:**

This single-center, retrospective cohort study enrolled patients undergoing unilateral, primary THA. A total of 197 patients (200 hips) were included in the final analyses, with 87 hips in the PAI group and 113 hips in the control group. Numeric Rating Scale (NRS) and laboratory data were assessed preoperatively and on postoperative days (POD) 1 and 7. Pearson’s correlation coefficients were obtained to assess the correlations between the D-dimer level on POD 7 and each outcome measure on POD 1.

**Results:**

The postoperative white blood cell count (WBC) was significantly higher in the PAI group than in the control group. Postoperative NRS, creatine phosphokinase (CK), and C-reactive protein (CRP) levels were significantly lower in the PAI group. D-dimer levels were significantly lower in the PAI group on POD 7. Postoperative aspartate transaminase (AST), alanine aminotransferase, blood urea nitrogen, and creatinine levels were within reference ranges. D-dimer levels on POD 7 showed a significant negative correlation with WBC on POD 1 (*r*=-0.4652) and a significant positive correlation with the NRS score and AST, CK, CRP, and D-dimer levels on POD 1 (*r* = 0.1558, 0.2353, 0.2718, 0.3545, and 0.3359, respectively).

**Conclusions:**

PAI containing a corticosteroid may be an effective treatment for pain and inflammation after THA, and it does not seem to cause drug-induced liver or kidney injury. Moreover, corticosteroid PAI can may accelerate early ambulation, which prevents the elevation of postoperative D-dimer levels, and may reduce the risk of deep venous thrombosis.

## Background

Total hip arthroplasty (THA) is a common, standardized, highly cost-effective surgical procedure [1]. However, earlier reports have found a patient dissatisfaction rate following THA of up to 11% [2]. Postoperative pain is an important factor affecting patients’ satisfaction with THA [2, 3]. Moreover, poorly managed postoperative pain can cause chronic postoperative pain [4]. Therefore, effective treatment of postoperative pain remains an important challenge for physicians [3]. Conventionally, many strategies have been applied to reduce postoperative pain, including peripheral nerve block, systemic morphine, and continuous epidural analgesia [5]. Recently, periarticular analgesic injection (PAI) has become a common alternative treatment for pain management following THA [6]. However, mixed opinions and conflicting results regarding PAI for pain management following THA have been reported [6–8]. PAI usually consists of local analgesics that may be combined with corticosteroids, opioids, epinephrine, or nonsteroidal anti-inflammatory drugs (NSAIDs) and then diluted with normal saline [7]. Several reports have been published regarding the analgesic effect of PAI containing corticosteroids and the anti-inflammatory effect of PAI containing NSAIDs in THA [9–11]. However, to the best of our knowledge, the systemic effects of PAI containing corticosteroids in patients subjected to THA have not been investigated.

In this study, we aimed to investigate the systemic effects of PAI containing corticosteroids and its efficacy for reducing postoperative pain in patients undergoing primary THA.

## Methods

This was a single center, retrospective cohort study. The study protocol adhered to the ethical guidelines of the 1975 Declaration of Helsinki, and the study was approved by the Ethics Committee Saga University Hospital. All patients provided opt-out informed consent prior to participation in this study.

A total of 254 patients (288 hips) who had undergone unilateral primary THA at our hospital between May 2019 and January 2020 were initially included. We implemented PAI at our hospital beginning in September 2019 for patients undergoing THA. Thus, patients who underwent THA from May 2019 to August 2019 were considered to be the control group, and patients who received THA between September 2019 and January 2020 comprised the PAI group. One hundred and forty patients (151 hips) were enrolled in the control group and 114 patients (137 hips) were enrolled in the PAI group. In the control group, we excluded 1 hip with hip ankylosis, 7 hips with femoral head necrosis, 5 hips with rapidly destructive coxarthrosis, 4 hips with osteotomy around the hip joints, 3 hips with post-traumatic arthritis of the hip joint, 2 hips with high hip dislocation, 1 hip with an intraoperative fracture, 2 hips with collagen diseases, 3 hips with medical complications, and 10 hips that lacked sufficient perioperative Numeric Rating Scale (NRS) data [12]. In the PAI group, we excluded 2 hips with hip ankylosis, 6 hips with femoral head necrosis, 2 hips with rapidly destructive coxarthrosis, 3 hips with osteotomy around the hip joint, 4 hips with post-traumatic arthritis of the hip joint, 5 hips with high hip dislocation, 2 hips with collagen diseases, 8 hips of patients with diabetes, and 18 hips that lacked sufficient perioperative NRS data. Finally, 197 patients (200 hips) with primary hip osteoarthritis or secondary hip osteoarthritis due to developmental dysplasia of the hip joint were enrolled. Thus, the analyses included a total of 113 hips (112 patients) in the control group and 87 hips (85 patients) in the PAI group (Table 1).

**Table 1:** Demographic data for the PAI and control groups

	Control group	PAI group	*P* value
Number of hips (patients)	113 (112)	87 (85)	
Sex (females), n (%)	95 (85)	77 (91)	0.4163
Age (years)	65.3 ± 8.8	65.9 ± 10.3	0.6784
BMI (kg/m^2^)	24.1 ± 3.2	24.2 ± 4.9	0.6117
Operation time (minutes)	47.8 ± 9.0	51.1 ± 19.9	0.4364
Intraoperative blood loss (g)	244.3 ± 90.0	248.6 ± 119.1	0.8815
Postoperative blood loss (g)	164.3 ± 133.1	149.0 ± 98.5	0.641

Values are expressed as the mean ± standard deviation. *PAI* periarticular analgesic injection, *BMI* body mass index

Anesthesia and surgery followed standardized procedures. All patients received spinal anesthesia with 0.5% isobaric bupivacaine in a single shot using a 27-gauge pencil-type spinal needle at the lower lumbar level. Midazolam (2–3 mg, intravenous injection) was administered for conscious sedation if needed. In all patients, 1 g of tranexamic acid and 1 g of cefazolin were administered intravenously before the skin incision to control surgical bleeding and prevent surgical site infection. All THA procedures were performed with a cementless femoral stem (910 PerFix or AG-PROTEX stem [Kyocera, Tokyo, Japan]) and an acetabular cup (910 PerFix or AG-PROTEX cup [Kyocera, Tokyo, Japan]) via a posterolateral approach. The suction drain was removed 1 day after surgery. In all patients, 1 g of cefazolin was administered intravenously before surgery in the operating room and three times within the time period between the patient’s return to the ward and the morning after surgery; and 30 mg of edoxaban tosilate hydrate was administered orally once per day from postoperative day (POD) 1 to POD 7.

In the PAI group, injections were performed after total hip prosthesis implantation and prior to closure. The PAI was a 41 ml solution containing 20 mL of 5 mg/mL levobupivacaine, l mL of 40 mg/mL triamcinolone acetonide (Kenacort-A® Intramuscular/Intraarticular Aqueous Suspension Injection; Bristol-Myers Squibb K.K., Tokyo, Japan), and 20 mL normal saline. The surgeon injected 10 mL of the solution into the capsule, 21 mL into the gluteus and external rotators, and 10 mL into the fatty layer.

The postoperative analgesic protocol was same for both groups. The patients received 50 mg of flurbiprofen axetil (Ropion**®**; Kaken Seiyaku Co., Ltd., Tokyo, Japan) as a continuous intravenous infusion within the first 24 h after surgery (total dose = 200 mg); acetaminophen (Acelio**®** Intravenous Injection; Terumo Corporation, Tokyo, Japan) at 1,000 mg for patients with body weight ≥ 50 kg (total dose = 4000 mg) or 15 mg/kg for patients with a body weight < 50 kg as an intravenous infusion every 6 h during the first 24 h after surgery; and celecoxib (Celecox**®**; Astellas Pharma Inc., Tokyo, Japan) 200 mg orally twice daily following an initial dose of 400 mg as the standard analgesic protocol. As rescue drugs, a 50 mg diclofenac sodium suppository (Voltaren**®** SUPPO**®**; Novartis Pharma K.K., Tokyo, Japan) or 15 mg of pentazocine (intramuscular) (Sosegon**®** Injection; Maruishi Pharmaceutical Co., Ltd., Tokyo, Japan) were administered.

Postoperative antithrombotic therapy was the same for both groups and included the following: edoxban (Lixiana ©, Daiichi Sankyo Company, Tokyo, Japan) for 7 days after surgery, wearing compression stockings during hospital stay, and early ambulation. The normal daily dose of endoxban was 30 mg (or 15 mg when patients weighed less than 50 kg; or 30 ml/min ≤ creatinine clearance ≤ 50 ml/min or 75 years and over) taken once orally. Walking training within the allowable pain range was started without weight-bearing limitations, beginning 1 day after surgery.

Sex, age, body mass index (BMI), operative time, intraoperative blood loss, and postoperative blood loss were assessed. Intraoperative blood loss was calculated based on the contents of the suction bottle and the change in the weight of the used surgical sponges. Postoperative blood loss was calculated based on the contents of the drain.

The primary outcome was the maximum pain level, assessed before surgery, on POD 1, and on POD 7. The patient’s pain level was assessed using the NRS. The NRS is a segmented numeric version of the visual analog scale in which a respondent selects a whole number (integers 0–10) that best reflects the intensity of their pain.

The secondary outcomes were the laboratory data obtained pre-surgery, on POD 1, and on POD 7, which were assessed using perioperative routine blood tests. Laboratory data included the white blood cell count (WBC), aspartate transaminase (AST), alanine aminotransferase (ALT), creatine phosphokinase (CK), blood urea nitrogen (BUN), creatinine (Cr), C-reactive protein (CRP), and D-dimer levels. Reference ranges for the laboratory data are as follows: WBC, 3300–9100/µl; AST, 10–35 U/l; ALT, 5–40 U/l; CK, 40–160 U/l; BUN, 8–20 mg/dl; Cr, 0.40–0.70 mg/dl; CRP, 0.00–0.30 mg/dl; and D-dimer, 0.00–1.00 µg/ml.

### Statistical analyses

All numerical data are expressed as the mean ± standard deviation. All analyses were performed using JMP® Pro software (version 14.2.0, SAS Institute Japan Ltd, Tokyo, Japan). We employed the Wilcoxson signed-rank test to compare BMI, operation time, intra- and postoperative blood loss, pre- and postoperative laboratory data, and pre- and postoperative NRS between the two groups. A Chi-squared test was used to compare the male:female proportion between the two groups. A Student’s *t*-test was used to compare the mean age between the two groups. The Steel-Dwass test was used to compare the perioperative data (NRS and laboratory data) within each group. Pearson’s correlation coefficients were obtained to assess the correlations between the D-dimer level on POD 7 and each outcome on POD 1. The level of significance was set at p < 0.05. Post-hoc analysis of the study was performed (effect size = 0.5, two-sided alpha = 0.05, sample size = 113 and 87), and the calculated power was 0.93.

## Results

There were no significant differences between the control and PAI groups in terms of age, sex, BMI, intra- or post-operative blood loss, or operation time (Table 1).

**Fig. 1:**
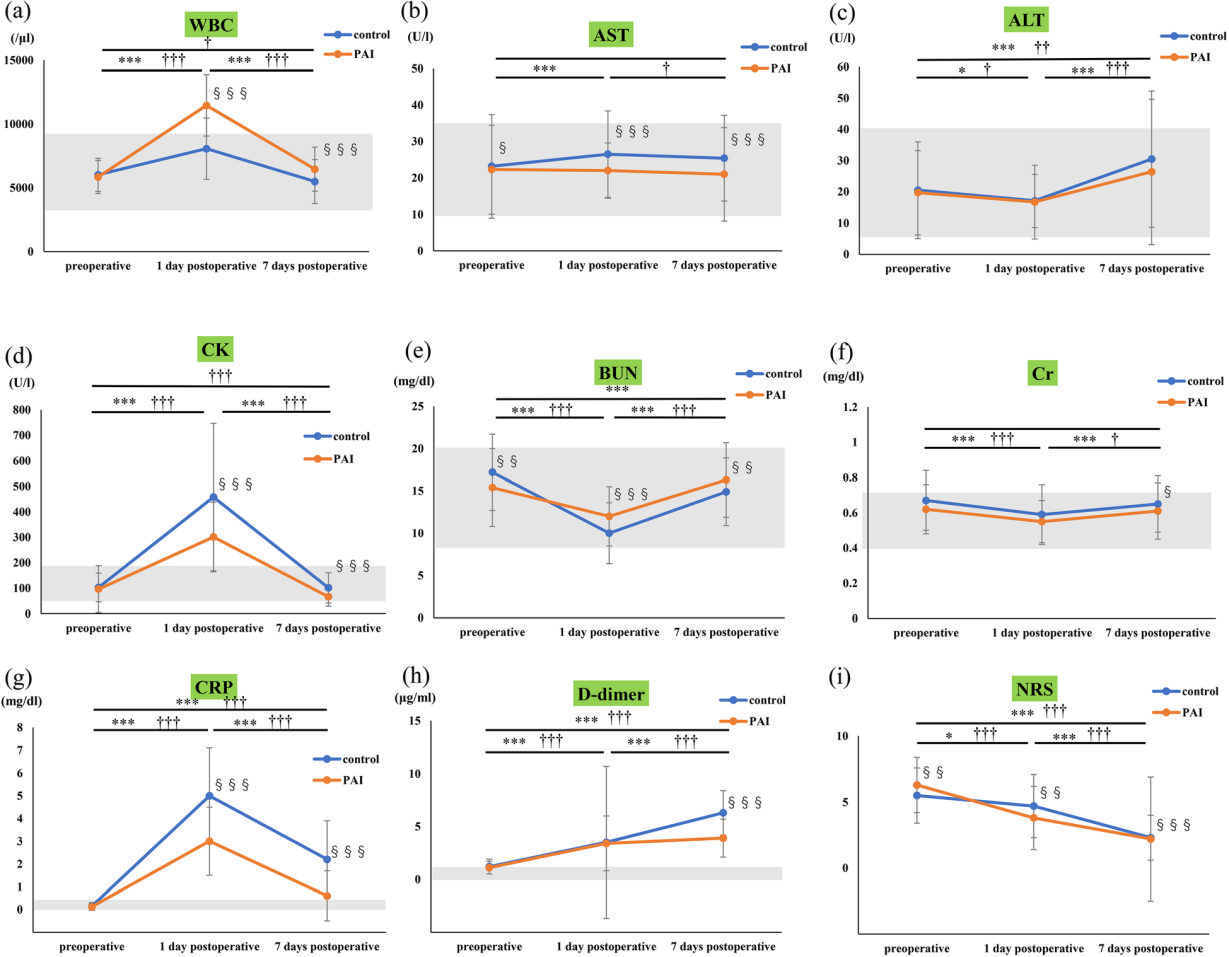
Comparison of perioperative data within each group and between the two groups. The gray zone shows the reference range for each laboratory value.Significant differences in perioperative data in the control group are marked as *P<0.05, **P<0.01, and ***P<0.001. Significant differences in perioperative data in the PAI group are marked as †*P*<0.05, ††*P*<0.01, and †††*P*<0.001. Significant differences between the two groups are marked as §*P*<0.05, §§*P*<0.01, and §§§*P*<0.001. PAI: periarticular analgesic injection, WBC: white blood cell count, AST: aspartate transaminase, ALT: alanine aminotransferase, CK: creatine phosphokinase, BUN: blood urea nitrogen, Cr: creatinine, CRP: C-reactive protein, NRS: numeric rating scale.

Preoperative NRS scores in the PAI group were significantly higher than those in the control group, whereas postoperative NRS scores in the PAI group were significantly lower than those in the control group (Table 2; Fig. 1i).

**Table 2:** Comparison of NRS scores between the PAI and control groups

	Control group (*N* = 113)	PAI group (*N* = 87)	*p* value
NRS scores			
Preoperative	5.5 ± 2.1	6.3 ± 2.1	0.004
1 day postoperative	4.7 ± 2.4	3.8 ± 2.4	0.0051
7 days postoperative	2.3 ± 1.7	2.2 ± 4.7	0.0114

Values are expressed as the mean ± standard deviation. *PAI* periarticular analgesic injection, *NRS* numeric rating scale

Table 3; Fig. 1a-h show the comparisons of laboratory values between the control and PAI groups and the comparison of perioperative laboratory data in each group. The postoperative WBC was significantly higher in the PAI group than in the control group, and within the PAI group the WBC was significantly higher on POD 7 than before surgery (Table 3; Fig. 1a) Postoperative CK levels were significantly lower in the PAI group than in the control group. Within the PAI group, CK levels were significantly lower on POD 7 than before surgery (Table 3; Fig. 1d). Postoperative CRP levels were lower in the PAI group than in the control group (Table 3; Fig. 1g). Seven days postoperatively, D-dimer levels were significantly lower in the PAI group than in the control group (Table 3; Fig. 1h). Postoperative AST, ALT, BUN, and Cr levels were within the reference ranges in both groups (Table 3; Fig. 1b, c, e and f).

**Table 3:** Comparison of laboratory data between the PAI and control groups

Laboratory data	Preoperative			POD 1			POD 7		
	Control group	PAI group	*p* value	Control group	PAI group	*p* value	Control group	PAI group	*p* value
	(*N* = 113)	(*N* = 87)		(*N* = 113)	(*N* = 87)		(*N* = 113)	(*N* = 87)	
WBC (/µl)	6019.4 ± 1653.6	5851.7 ± 1299.5	0.7421	8072.6 ± 1654.2	11470.1 ± 2409.0	< 0.0001	5491.2 ± 1274.9	6464.4 ± 1726.4	< 0.0001
AST (U/l)	23.3 ± 14.2	22.3 ± 12.2	0.0477	26.5 ± 11.9	22.0 ± 7.6	< 0.0001	25.4 ± 11.8	21 ± 12.8	< 0.0001
ALT (U/l)	20.5 ± 15.5	19.7 ± 13.5	0.2959	17.1 ± 8.5	16.7 ± 11.8	0.0653	30.5 ± 21.8	26.4 ± 23.3	0.0766
CK (U/l)	102.6 ± 56.4	96.1 ± 92.2	0.0584	457.8 ± 289.2	301.1 ± 136.7	< 0.0001	101.2 ± 59.0	66.0 ± 36.8	< 0.0001
BUN (mg/dl)	17.2 ± 4.5	15.4 ± 4.6	0.005	10.0 ± 3.6	12.0 ± 3.5	< 0.0001	14.9 ± 4.0	16.3 ± 4.4	0.0091
Cr (mg/dl)	0.67 ± 0.17	0.62 ± 0.14	0.069	0.59 ± 0.17	0.55 ± 0.12	0.0742	0.65 ± 0.16	0.61 ± 0.16	0.0281
CRP (mg/dl)	0.14 ± 0.17	0.12 ± 0.15	0.7851	5.0 ± 2.1	3.0 ± 1.5	< 0.0001	2.2 ± 1.7	0.6 ± 1.1	< 0.0001
D-dimer (µg/ml) (【(µg/mL)	1.2 ± 0.7	1.1 ± 0.6	0.3573	3.5 ± 7.2	3.4 ± 2.6	0.5273	6.3 ± 2.1	3.9 ± 1.8	< 0.0001

Values are expressed as the mean ± standard deviation. *PAI* periarticular analgesic injection, *POD* postoperative day, *WBC* white blood cell count, *AST* aspartate transaminase, *ALT* alanine aminotransferase, *CK* creatine phosphokinase, *BUN* blood urea nitrogen, *Cr* creatinine, *CRP* C-reactive protein

In all hips, combining both groups, D-dimer levels on POD 7 had a significant negative association with the WBC on POD 1 (*r*=-0.4652; *p* < 0.0001). D-dimer levels on POD 7 had a significant positive association with the NRS score and AST, CK, CRP, and D-dimer levels on POD 1 (*r* = 0.1558, 0.2353, 0.2718, 0.3545, and 0.3359, respectively; Table 4).

**Table 4:** Correlations between D-dimer levels on POD 7 and data on POD 1 (*N* = 200)

		95% CI		
	*r*	Lower	Upper	*p* value
NRS	0.1558	0.0175	0.2884	0.0275
WBC	-0.4652	-0.5673	-0.3490	< 0.0001
AST	0.2353	0.0998	0.3622	< 0.0001
ALT	0.0525	-0.0869	0.1899	0.4604
CK	0.2718	0.1382	0.3956	< 0.0001
BUN	-0.1694	-0.3010	-0.0314	0.0166
Cr	-0.0284	-0.1665	0.1108	0.6899
CRP	0.3545	0.2269	0.4701	< 0.0001
D-dimer	0.3359	0.2068	0.4535	< 0.0001

*POD* postoperative day, *PAI* periarticular analgesic injection, *CI* confidence interval, *NRS* numeric rating scale, *WBC* white blood cell count, *AST* aspartate transaminase, *ALT* alanine aminotransferase, *CK* creatine phosphokinase, *BUN* blood urea nitrogen, *Cr* creatinine, *CRP* C-reactive protein

## Discussion

To the best of our knowledge, this study is the first to investigate the effect of PAI containing a corticosteroid on laboratory data following THA. Our findings indicate that corticosteroid PAI is an effective treatment for pain and inflammation after THA. Corticosteroid PAI may lead to early ambulation, reduced D-dimer levels, and, consequently, a reduced risk of deep venous thrombosis (DVT).

Levobupivacaine, which is the S-enantiomer of bupivacaine, is a long-acting local anesthetic drug [13]. Compared to ropivacaine, levobupivacaine has a longer duration of action and the half-life is approximately 4 h [13–15]. Levobupivacaine has a wider margin of safety, in terms of cardiovascular and central nervous system adverse effects, compared with bupivacaine [16]. Therefore, levobupivacaine has been used in PAI [17, 18]. However, some previous studies have found that PAI (levobupivacaine and/or epinephrine) in THA did not reduce postoperative pain [17, 18]. Conversely, our study demonstrated lower NRS scores in the PAI group on PODs 1 and 7, which indicates that the corticosteroid may have had an effect on postoperative pain.

Previous studies reported that trauma and surgery, including THA, cause the release of interleukin (IL)-6, causing local inflammation at the site of injury [19–21]. CRP is an acute-phase protein that increases after inflammation and is a marker that reflects the severity of inflammation [19, 22]. The release of IL-6 during inflammation leads to the production of CRP [19]. CK is an energy production enzyme in the mitochondria and is primarily found in muscle tissues [23]. CK elevation is a feature of muscle inflammation or damage [24]. Corticosteroids have an anti-inflammatory effect, via the inhibition of the synthesis of phospholipase A2, thereby reducing the production of the pro-inflammatory derivatives of arachidonic acid, e.g. IL-1, IL-2, IL-6, and tumor necrosis factor (TNF-α) [25, 26]. An anti-inflammatory effect of glucocorticoids (e.g., reduction of IL-6 in the drain fluid and CRP) in total knee arthroplasty has been reported [27]. Postoperative CRP and CK levels were lower in the PAI group than in the control group, owing to the anti-inflammatory effect of the corticosteroid in this study.

Triamcinolone acetonide was the corticosteroid used in this study, which is an intermediate-acting glucocorticoid with a half-life between 18 and 36 h [25, 28]. Intramuscular administration of triamcinolone acetonide provides a slow absorption and prolonged duration [28, 29]. In this study, CRP levels and CK levels on POD 7 were lower in the PAI group than in the control group. Surprisingly, in the PAI group, CK levels were significantly lower on POD 7 than before surgery. Therefore, PAI containing triamcinolone acetonide may have a prolonged anti-inflammatory effect after THA, for at least 7 days postoperatively.

Postoperative pain is considered a form of acute pain owing to surgical trauma with an inflammatory reaction and initiation of an afferent neuronal barrage [30]. Therefore, reducing inflammation is important for reducing postoperative pain. Additionally, postoperative inflammation affects the immediate functional recovery after THA [31], and pain management following THA is important for early postoperative rehabilitation [32]. This study demonstrated the analgesic effect of PAI containing a corticosteroid on POD 7 owing to the anti-inflammatory effect of a corticosteroid that has a long duration of activity [28, 29]. Therefore, the analgesic effect of PAI containing a corticosteroid may accelerate early postoperative rehabilitation.

DVT and pulmonary embolus are potential life-threating complications after THA, and their prevention is a universal quality improvement initiative [33, 34]. D-dimer is a degradation product of a cross-linked fibrin blood clot [35], and the elevation of D-dimer levels is caused by acute venous thromboembolism, recent major surgery, hemorrhage, trauma, pregnancy, and cancer [35]. A D-dimer test is a simple and useful method for diagnosing DVT, because the incorporation of a D-dimer test into a clinical diagnostic strategy can identify DVT without ultrasonography [36, 37]. Antithrombotic drugs, an intermittent compression device, pneumatic foot pumps, graduated compression stockings, and early ambulation are methods for preventing DVT in patients after surgery [38, 39]. Additionally, early ambulation prevents the development of high postoperative D-dimer levels [40]. In our study, walking training began after POD 1 in both groups; thus, early ambulation had no effect on the D-dimer levels on POD 1. Rather, D-dimer levels on POD 1 were only affected by the surgery [35]. Hence, there was no significant difference between the D-dimer levels of the two groups on POD 1. On the other hand, the analgesic and anti-inflammatory effects of corticosteroid PAI from POD 1 may accelerate early ambulation. Therefore, the D-dimer levels were significantly lower in the PAI group than in the control group on POD 7.

Glucocorticoids can stimulate the bone marrow to produce more granulocytes, inhibit neutrophil apoptosis, and impair the migration of granulocytes to sites of inflammation or infection through the vasculature [41–43]. This results in increased numbers of circulating neutrophils [41–43]. In this study, the postoperative WBC was significantly higher in the PAI group than in the control group, and the WBC in the PAI group was significantly higher on POD 7 than before surgery. The postoperative WBC elevation in the PAI group may be due to the prolonged effect of the corticosteroid on granulocytes. Our results were contradictory because the postoperative WBC increased and the postoperative CRP levels decreased in the PAI group, even though WBC and CRP are both markers of inflammation [19, 22, 44]. The effect of increased number of circulating neutrophils may be greater than the effect of lowering WBC by anti-inflammation using the corticosteroid.

AST is found in the liver, heart, muscle, kidneys, brain, and blood cells, [45] while ALT is found in the plasma and other organs; however, most ALT is found in the liver [45].

In our study, postoperative AST levels were lower in the PAI group than in the control group. There were no significant differences between the pre and postoperative AST levels within the PAI group. Postoperative ALT levels were not significantly different between the two groups. Levobupivacaine and corticosteroids are mainly metabolized by the liver [46, 47]; normal postoperative ALT levels in both groups indicate that there was no drug hepatopathy following THA with or without corticosteroid PAI. Thus, the anti-inflammatory effects of the corticosteroid may have prevented muscle damage and influenced AST levels in the PAI group, as observed with CK.

BUN and Cr are biomarkers of kidney function [48]. The differences in BUN and Cr levels on POD 7 were inconsistent between the groups. Both BUN and Cr levels were within their reference ranges on POD 7. The metabolization of levobupivacaine and corticosteroids occurs primarily in the liver [46, 47]; therefore, it was assumed that the PAI containing a corticosteroid did not directly produce any drug-induced kidney injury.

In the PAI group, the NRS score, CPK, CRP levels on POD 1 were lower than those in the control group, which reflects the analgesic and anti-inflammatory effects of the PAI containing a corticosteroid. In addition, pain management and reducing inflammation following THA are important for early postoperative rehabilitation as they can prevent the elevation of postoperative D-dimer levels and reduce the risk of DVT [31, 32, 39, 40]. In all hips in both groups, the NRS score, CPK, and CRP levels on POD 1 showed significant positive correlations with D-dimer levels on POD 7. Therefore, a PAI containing a corticosteroid is a useful method for pain management and for reducing inflammation following THA. D-dimer levels tend to rise after surgery, and the half-time is quite long [35, 49]. Hence, the D-dimer levels on POD 1 may have affected the D-dimer levels on POD 7, perhaps resulting in the significant positive correlation between D-dimer levels on POD 1 and those on POD 7 in this study. As for the WBC, in the PAI group, the WBC on POD 1 was higher than that in the control group because the WBC on POD 1 was elevated, owing to the effect of the corticosteroid on circulating neutrophils. The D-dimer levels on POD 7 in the PAI group were lower than those in the control group, likely due to early ambulation. These may result in the significant negative correlation between the WBC on POD 1 and D-dimer levels on POD 7.

There are several limitations in our study. First, CRP was the only inflammatory marker investigated. Other indices to evaluate inflammation, such as IL-6, were lacking. However, evaluating CRP may be sufficient for investigating the grade of inflammation because CRP is highly correlated with IL-6 [50]. Second, postoperative functional performance was not investigated. Therefore, we could not investigate an association between postoperative functional performance and D-dimer levels. In the future, assessments of postoperative functional performance are needed. Third, there was no assessment of the incidence of DVT, which is associated with elevated D-dimer levels, using computed tomographic scanning or ultrasonography [51]. Although imaging tests are not always necessary for the diagnosis of DVT [37], imaging tests may be needed to determine whether PAI containing a corticosteroid accurately reduces DVT in future studies. Finally, the contents of the PAI in this study were a corticosteroid and levobupivacaine. A group that receives a PAI containing only a corticosteroid, without levobupivacaine, may be necessary to definitively investigate the anti-inflammatory effect of corticosteroids.

## Conclusions

PAI containing a corticosteroid was found to be an effective treatment for pain and inflammation following THA. Considering the lower postoperative D-dimer levels observed in the PAI group, the analgesic and anti-inflammatory effects of corticosteroid PAI may have the potential to accelerate early ambulation and reduce the risk of DVT.

## Data Availability

The datasets used and/or analyzed during the current study are available from the corresponding author on reasonable request.

## Abbreviations

ALT: Alanine aminotransferase
AST: Aspartate transaminase
BMI: Body mass index
BUN: Blood urea nitrogen
CK: Creatine phosphokinase
Cr: Creatinine
CRP: C-reactive protein
DVT: Deep venous thrombosis
IL: Interleukin
NRS: Numeric Rating Scale
NSAID: Nonsteroidal anti-inflammatory drugs
PAI: Periarticular analgesic injection
POD: Postoperative day
THA: Total hip arthroplasty
TNF: Tumor necrosis factor
WBC: White blood cell count
